# Accelerometer‐Determined Physical Activity and Sarcopenic Obesity Risk in Older European Men and Women

**DOI:** 10.1002/jcsm.70149

**Published:** 2025-12-04

**Authors:** Andreas Nilsson, Hadil Limem, Aurelia Santoro, Laura Smeldy Jurado‐Medina, Agnes A. M. Berendsen, Lisette C. P. G. M. de Groot, Joanna Kaluza, Ewa Sicińska, Amy Jennings, Susan Fairweather‐Tait, Alberto Bazzocchi, Giuseppe Battista, Claudio Franceschi, Tarak Driss, Fawzi Kadi

**Affiliations:** ^1^ School of Health Sciences Örebro University Örebro Sweden; ^2^ Interdisciplinary Laboratory in Neurosciences, Physiology, and Psychology: Physical Activity, Health, and Learning (LINP2), UFR STAPS Paris Nanterre University Paris by Nanterre France; ^3^ Department of Medical and Surgical Sciences University of Bologna Bologna Italy; ^4^ Interdepartmental Centre “Alma Mater Research Institute on Global Challenges and Climate Change (Alma Climate)” University of Bologna Bologna Italy; ^5^ Division of Human Nutrition Wageningen University & Research Wageningen the Netherlands; ^6^ Department of Human Nutrition Warsaw University of Life Sciences (WULS‐SGGW) Warsaw Poland; ^7^ Co‐Centre for Sustainable Food Systems & Institute for Global Food Security, Queens University Belfast Belfast UK; ^8^ Norwich Medical School University of East Anglia Norwich UK

**Keywords:** adiposity, ageing, low intensity physical activity, metabolic risk, moderate‐to‐vigorous physical activity, sarcopenia

## Abstract

**Background:**

Sarcopenic obesity (SO) is characterized by the presence of both obesity and sarcopenia and is related to disability and loss of independence in older adults. The extent to which time spent in light physical activity (LPA), or moderate‐to‐vigorous physical activity (MVPA) is associated with SO risk in older adults remains unclear. The aim of this study was (a) to examine the association between the level of adherence to recommended amounts of MVPA and the risk of SO in older adults and (b) to determine whether time spent in LPA is associated with SO risk independently of time spent in MVPA.

**Methods:**

This cross‐sectional study involved 862 community‐dwelling older adults (58% women; aged 65–79 years) from four European countries. Accelerometer‐determined time in MVPA was categorized as follows: inactive (< 75 min/week), moderately active (75–149 min/week), active (150–299 min/week) and highly active (≥ 300 min/week). Time in LPA was expressed in tertiles. The outcome measure SO risk was determined based on appendicular lean mass, waist circumference, handgrip strength and the 5‐times sit‐to‐stand test. Odds ratios (OR) with a 95% confidence interval (95% CI) of high SO risk across levels of MVPA and LPA were determined by binary logistic regression adjusted for the level of systemic inflammation (high‐sensitivity C‐reactive protein) and dietary protein intake.

**Results:**

Compared to the inactive group, ORs of having a high SO risk were about 50%–80% lower, depending on the MVPA level, with the largest risk reduction in the highly active group (OR: 0.23, 95% CI: 0.13–0.39; *p* < 0.05). The likelihood of having a high SO risk was significantly lower among the highly active group compared to the active group (OR: 0.50; 95% CI: 0.33–0.77; *p* < 0.05). More time in LPA was associated with a significantly lower likelihood of having high SO risk (highest vs. lowest tertile: OR: 0.52, 95% CI: 0.30–0.89; *p* < 0.05) only in participants with low amounts of MVPA. In contrast, LPA was not associated with SO risk among participants meeting the MVPA recommendation.

**Conclusions:**

MVPA is strongly associated with a lower likelihood of having a high SO risk in older adults, independently of the level of systemic inflammation and intakes of dietary proteins. LPA is related to SO risk in sedentary older adults, which supports the promotion of physical activity regardless of intensity for mitigating SO.

## 1 Introduction

While sarcopenia describes an age‐related condition of accelerated loss of muscle mass and impaired muscle function, the notion that a parallel accumulation of body fat may exacerbate muscle wasting by metabolic alterations has called for the definition of sarcopenic obesity (SO), a clinical condition that should be viewed separately from sarcopenia alone [1]. Interventions targeting lifestyle behaviours, including calorie restriction and diet quality alongside regular physical activity are considered key elements in the management of SO [2]. Although the exact mechanisms explaining the detrimental interplay between increased adiposity and muscle wasting remain unclear, gains in adipose tissue—particularly visceral fat deposits—are associated with elevated levels of pro‐inflammatory biomarkers, including C‐reactive protein, which have been shown to promote accelerated loss of muscle mass [2, 3]. Furthermore, elevated levels of intramyocellular lipids have been hypothesized to cause elevated oxidative stress and decreased insulin sensitivity, negatively affecting the normal metabolic and cellular function of skeletal muscle [2, 4]. Therefore, SO, characterized by the presence of both obesity and sarcopenia, promotes an accelerated decline in physical function towards frailty, disability and loss of independence in older adults [1, 2].

Physical activity (PA) is commonly regarded as a key lifestyle factor to promote the maintenance of physical function alongside mitigating the accumulation of adiposity. Therefore, current global guidelines for healthy ageing recommend engagement in aerobic moderate‐to‐vigorous PA (MVPA), with a target range between 150 and 300 weekly minutes to minimize the risk of injuries, alongside engagement in muscle‐strengthening activities (MSA) to improve or maintain physical function [5]. Indeed, lower prevalence rates of SO have been reported in older adults who regularly engage in MVPA compared to those less active in several [6, 7, 8, 9, 10] but not all studies [11]. Importantly, most previous studies have used self‐report instruments to assess amounts or patterns of PA [6, 7, 8, 11], which are prone to recall bias and have less ability to provide detailed information about time spent in PA of different intensities compared to objective methodologies [12]. This may have implications for efforts to explain the true role of PA behaviours, including time spent sedentary or in light intensity PA (LPA), in the development of SO in older adults.

The current PA guideline advocates the accumulation of 150 to 300 weekly minutes of MVPA as an appropriate target range for health benefits, and increasing the amount above this target range may be associated with additional health benefits [5]. However, the extent to which accumulating MVPA beyond this range (i.e., ≥ 300 min/week) will provide additional impacts on SO risk is yet to be clarified in older adults. Furthermore, a previous meta‐analysis based on objectively assessed time in MVPA in adult populations indicated that a significantly reduced mortality risk was evident in adults spending half the minimum recommended amount of weekly MVPA (75 min) compared to those spending less time [13]. Therefore, it may be hypothesized that time spent in MVPA even below the minimum recommended amount (e.g., 75 to 150 min/week) is related to a lower risk of SO when compared to less active individuals (< 75 min/week). Moreover, besides MVPA, time spent in LPA may also have the potential to positively influence SO and its components. For example, we have recently shown that less daily time spent sedentary in favour of more time in LPA is related to lower metabolic syndrome risk in older adults, independently of adherence to the MVPA guideline [14]. To date, very few studies have examined the influence of objectively assessed time in LPA on SO specifically. While one study indicated a significant impact of time spent in LPA on SO [10], another study based on a 2‐day measure of PA did not find such an effect in older adults [9]. Therefore, the potential of LPA to confer a beneficial impact on SO risk in older adults, and whether this impact is independent of time spent in MVPA, remains to be clarified.

An important limitation typical of most previous studies is that the intake of dietary proteins has not been considered when analysing the role of PA behaviours on SO. This is unfortunate as dietary proteins are anabolic factors involved in the maintenance of muscle mass and strength [15] and are demonstrated to be associated with components of sarcopenia in older adults [16]. Furthermore, adherence to the MVPA guideline has been linked to higher adherence to healthy eating habits, including higher intakes of dietary proteins [17], which emphasizes the need to consider the effects of dietary proteins in order to disentangle the true influence of PA behaviours on SO.

A better understanding of the extent to which time spent in different intensities of PA is associated with SO risk in older adults holds important clinical implications in terms of both prevention and management of SO. In light of this knowledge gap, the primary aim of the present study was to examine the extent to which different levels of objectively measured adherence to the MVPA guideline are associated with the risk of SO in older adults, while considering important factors such as dietary protein intake and level of systemic inflammation. The secondary aim was to determine whether time spent in LPA is associated with SO risk independently of adherence to the MVPA guideline.

## 2 Methods

### 2.1 Study Design and Participants

The present study is based on cross‐sectional analyses of data from the NU‐AGE database, comprising a wide array of variables covering socio‐demographic, medical, biological and lifestyle‐related outcomes collected from populations of European older adults as previously described in detail [18, 19]. Participants received oral and written information about the study and signed a written informed consent. All study procedures were conducted in accordance with the Declaration of Helsinki. The study was approved by local ethical review boards: Independent Ethics Committee of the Sant'Orsola—Malpighi Hospital Bologna (Italy‐03/2011/U/Sper), Bioethics Committee of the Polish National Food and Nutrition Institute (decision of 03.04.2012), the Wageningen University Medical Ethics Committee (Netherlands‐11/41) and the National Research Ethics Committee East of England (UK‐12/EE/0109). For the present study, data from four NU‐AGE research centres (Bologna in Italy, Warsaw in Poland, Wageningen in the Netherlands and Norwich in the United Kingdom) were used, including PA behaviours assessed by hip‐worn accelerometers. The sample of older men and women (*n* = 1020) included in the NU‐AGE database was originally recruited to ensure a statistical power of 80% (alpha 5%) to detect clinically relevant changes in C‐reactive protein after a dietary intervention [19]. Criteria for inclusion were: age between 65 and 79 years, non‐frail community‐dwelling, competent to make own decisions. Exclusion criteria were overt diseases such as type 2 diabetes, cancers, dementia, organ failure and history of severe heart disease and the presence of frailty. In addition, as the present study aimed to examine the likelihood of developing SO (low vs. high SO risk), participants fulfilling diagnostic criteria for SO (*n* = 3) at screening were excluded. The present study follows the Strengthening the Reporting of Observational Studies in Epidemiology (STROBE) reporting guidelines [20].

### 2.2 Measurements

#### 2.2.1 PA Behaviours

Time spent in PA of different intensities was objectively assessed for a week using the Actigraph GT3x accelerometer (Actigraph, Pensacola, Florida). Participants wore the monitor on the right hip, tightly secured with an elastic belt during all waking hours, except during water activities. At least 4 days with at least 10 h of wear time per day were required for inclusion in the present analysis. A minimum of 90 min of continuous zero counts was defined as non‐wear time [21]. Time spent in MVPA (≥ 2020 counts per minute), LPA (100–2019 counts per minute) and sedentary behaviours (SB) (< 100 counts per minute) was determined based on established accelerometer count cut‐points [22, 23]. Participants were categorized according to level of adherence to the MVPA guideline as follows: inactive (< 75 min/week), moderately active (75–149 min/week), active (150–299 min/week) and highly active (≥ 300 min/week). Daily accumulated time spent in LPA was expressed in relation to total monitor wear time (%) and categorized based on mathematically derived tertiles labelled as low (Tertile 1), medium (Tertile 2) and high (Tertile 3). In addition, weekly engagement in muscle‐strengthening activities (MSA) was self‐reported using the validated Physical Activity Scale for the Elderly (PASE) [24].

#### 2.2.2 Anthropometrics

Body height (cm) was recorded with a stadiometer to the nearest 0.5 cm, and body weight (BW) was measured in light clothing to the nearest 0.1 kg using a calibrated scale. Waist circumference (WC) was determined midway between the lowest rib and the iliac crest to the nearest 0.1 cm with the participant standing relaxed in an upright position. Appendicular lean mass (ALM) was assessed using whole‐body dual‐energy X‐ray absorptiometry (DXA) (Lunar iDXA, GE Healthcare, Madison, WI, USA—enCORETM 2011 software version 13.6, Bologna, Italy; Lunar Prodigy, GE Healthcare, Madison, WI, USA—enCORETM 2011 software version 13.6, Wageningen, the Netherlands, Warsaw, Poland; and Discovery Wi, Hologic Inc., Bedford, MA, USA, Norwich, UK). Procedures for DXA scans on this cohort of older adults have previously been reported elsewhere [25]. In brief, participants were scanned in a supine position, with their arms separated from the trunk, and metal items were removed before scanning.

#### 2.2.3 Biomarker of Systemic Inflammation

Blood samples were taken after an overnight fast, where participants were instructed to refrain from alcohol and heavy exercise in the prior 24 h. After 30 min at room temperature, blood samples were centrifuged at 2000 × *g* for 10 min at 4°C and serum was collected. The clinically established inflammatory biomarker high sensitivity C‐reactive protein (hsCRP) was determined using ProcartaPlex Immunoassay (Thermo Fisher Scientific) in accordance with the manufacturer's instructions. The analysis was performed using Luminex 200 instrumentation (Luminex Corporation). Participants were classified as having a low, intermediate, or high level of systemic inflammation based on the following hsCRP levels: < 1, 1–2.99 or ≥ 3 mg/L, respectively [26].

#### 2.2.4 SO Risk

High SO risk was determined based on the SO components presented in the consensus statement on definitions and diagnostic criteria issued by the European Society for Clinical Nutrition and Metabolism (ESPEN) and the European Association for the Study of Obesity (EASO) [1]. Accordingly, the coexistence of abdominal obesity determined by WC, low muscle strength determined by (a) handgrip (HG) strength using the Jamar handheld dynamometer (Patterson Medical, Warrenville, IL, USA) and (b) the 5‐times sit‐to‐stand (5‐STS) test, and low muscle mass determined by DXA‐derived ALM expressed in relation to BW (%BW) were required to be classified as having high SO risk. We classified participants as having high SO risk based on the following three‐step procedure: (1) WC above the ethnic cut‐point for abdominal obesity according to the International Diabetes Federation criteria [27], (2) being below the sex‐specific median value for HG or above the median value for 5‐STS and (3) being below the sex‐specific median value for ALM (%BW). The presence of abdominal obesity in step 1 was a prerequisite to continue to step 2 to ensure the coexistence of abdominal obesity with higher sarcopenia risk according to the SO definition [1]. The sex‐specific cut points for the SO risk components used in the present study are shown in Table S1.

#### 2.2.5 Covariates

Information on medical history, including disease/conditions related to musculoskeletal health (osteoporosis, osteoarthritis, hip fracture and falls last 12 months), general characteristics, including years of education, marital status and smoking status, were self‐reported. General health status was assessed based on the 36‐item Short Form (SF‐36) questionnaire. Dietary protein intake and alcohol consumption were determined using seven‐day preformatted food records as previously described [28]. In brief, prior to recording, instructions about how to describe the amounts and types of foods consumed were given to participants by trained dieticians at each recruiting centre. All food records were coded and translated into nutrients using local food composition tables in each recruiting centre (BDA and INRAN in Italy, NFNI in Poland, NEVO 2011 in the Netherlands, McCance and Widdowson's in the United Kingdom) and softwares (DIETA‐5, National Food and Nutrition Institute, Warsaw, Poland; Compl‐eat, Wageningen university & research, Wageningen, the Netherlands; NetWISP v4.0, Tinuviel Software, Warrington, UK). Participants were categorized into having high‐ or low‐risk alcohol consumption based on a threshold of 20 and 10 g/day for men and women, respectively, in accordance with previous work [28]. A daily protein intake of ≥ 1.1‐g/kg BW was used to determine adherence to the recommended daily protein intake for older adults [29].

### 2.3 Statistical Analyses

Data are presented as mean and standard deviation or as percentages. Differences in study characteristics between groups of SO risk were determined by independent‐samples *t*‐test (age and education years) or chi‐square (*χ* ^2^) analysis (categorical variables). The likelihood of having a high SO risk across different PA behaviours was determined using binary logistic regression analysis with a conditional backward elimination model, with the probability threshold for variable removal set to *p* > 0.1. Analysis of SO risk across the four categories of weekly MVPA (inactive, moderately active, active and highly active) was adjusted by the following covariates: age, sex, study centre, marital status (married/unmarried), education years, smoking habits (never, former and current), general health status (poor, fair, good and excellent), hsCRP level (low, intermediate and high), number (0, 1 or ≥ 2) of disease/conditions related to musculoskeletal health, engagement in MSA (yes/no), adherence to recommended protein intake (yes/no), adherence to low‐risk consumption of alcohol (yes/no), daily SB time (min) and accelerometer wear time (min). This analysis was performed with the inactive group set as reference, and additionally by comparing each MVPA category with the next ascending category set as reference (e.g., Active vs. Highly active). We also analysed the likelihood of having a high SO risk across tertiles of daily LPA time stratified by adherence to the recommended minimum amount of weekly MVPA time and with the low LPA tertile set as reference. Daily SB time was replaced by MVPA time as a covariate to control for potential confounding from engagement in PA above the light intensity threshold. In addition, the impact of reallocating 30‐min time blocks spent in LPA with corresponding time blocks of SB or MVPA, while holding total monitor wear time constant, on SO risk was analysed after adjustment for covariates described above. Finally, to clarify the role of PA intensity on SO risk, we analysed the impact of reallocating a fixed PA volume block (20 000 daily accelerometer counts approximately corresponding to 10 min at the MVPA intensity threshold) derived from MVPA, with an equal block derived from LPA, while holding total PA volume constant. Results are presented as odds ratios (OR) with 95% confidence intervals (95% CI). All analyses were performed using SPSS statistics, version 29 (Armonk, NY, USA: IBM Corp), and the level of significance was set to *p* < 0.05.

## 3 Results

### 3.1 Participant Characteristics

Out of the 1020 older adults recruited for inclusion, 158 were excluded due to incomplete data for the present analyses, yielding a final study sample of 862 (mean age 71 ± 4 years) community‐dwelling men (*n* = 361) and women (*n* = 501). Thirty‐four percent of the whole sample fulfilled the study criteria for high SO risk. PA behaviours were monitored for an average of 14.6 ± 0.9 h per day, where an average of 8.8 ± 1.3 and 5.3 ± 1.2 h/day were spent in SB and LPA, respectively, and with a remaining 27.7 ± 21.2 min/day spent in MVPA. Based on MVPA time, 21%, 26%, 32% and 21% of the participants were classified as inactive, moderately active, active and highly active, respectively.

Study characteristics stratified by groups of low and high SO risk are presented in Table 1. Compared to the low SO risk group, the high SO risk group was characterized by a significantly higher prevalence (*p* < 0.05) of elevated hsCRP, poor general health status, osteoarthritis and hip fracture. In addition, a significantly (*p* < 0.05) larger proportion of high SO risk participants did not report engagement in MSA and did not adhere to guidelines for either MVPA or protein intake compared to their low SO risk peers (Table 1).

**TABLE 1:** Study characteristics in participants with low and high sarcopenic obesity (SO) risk.

	Low SO risk (*n* = 573)	High SO risk (*n* = 289)
Age (years)	70.6 ± 3.9	71.6 ± 4.1*
Sex (% women)	56.4	61.6
Marital status (% married)	67.9	66.4
Education (years)	12.8 ± 3.4	12.6 ± 3.9
hsCRP categories (%)		
Low (< 1 mg/L)	61.6	42.2*
Intermediate (1–2.99 mg/L)	27.4	40.5
High (≥ 3 mg/L)	11.0	17.3
General health status (%)		
Excellent	20.2	10.7*
Good	25.3	21.5
Fair	33.7	31.5
Poor	20.8	36.3
Osteoporosis (%)	10.6	12.5
Osteoarthritis (%)	32.6	37.7*
Hip fracture (%)	2.1	3.5*
Falls last 12 months (%)	14.0	14.9
Smoking (%)		
Never	53.9	49.5
Former	41.7	46.0
Current	4.4	4.5
Adherence to low‐risk alcohol consumption (%)	73.5	76.5
Adherence to the protein guideline (%)	42.1	19.0*
MSA (% yes)	19.4	9.7*
Adherence to the MVPA guideline (%)	59.2	39.8*

Abbreviations: BW, body weight; hsCRP, high‐sensitivity C‐reactive protein; MSA, muscle‐strengthening activities; MVPA, moderate‐to‐vigorous physical activity.

* *p* < 0.05.

Data on components of SO risk in participants classified as having low or high SO risk are shown in Table 2. The sex‐specific criteria used to classify participants into low or high SO risk resulted in differences in all SO risk components, varying from 10% to 25% with higher average WC and 5‐STS time and lower ALM and HG in the high SO risk compared to the low SO risk group (Table 2).

**TABLE 2:** Components of sarcopenic obesity in men and women classified with low or high sarcopenic obesity (SO) risk.

	Low SO risk		High SO risk	
	Men (*n* = 250)	Women (*n* = 323)	Men (*n* = 111)	Women (*n* = 178)
Waist circumference (cm)	94.2 ± 9.6	84.3 ± 9.6	105.7 ± 8.5	94.4 ± 9.1
ALM (%BW)	32.0 ± 2.9	25.8 ± 2.5	28.1 ± 1.7	22.2 ± 1.6
HG (kg)	41.2 ± 7.2	26.8 ± 5.7	38.1 ± 6.9	23.4 ± 5.0
5‐STS (s)	9.1 ± 2.2	10.0 ± 2.7	11.0 ± 2.4	11.8 ± 3.2

Abbreviations: 5‐STS, 5‐times sit‐to‐stand test; ALM, appendicular lean mass; BW, body weight; HG, handgrip.

### 3.2 SO Risk Across Time Spent in Different PA Behaviours

We first examined the impact on SO risk by reallocating 30‐min time blocks spent in LPA with corresponding time blocks of SB or MVPA after adjustment for covariates. The analysis showed that replacing LPA with SB was related to a significantly higher likelihood of having high SO risk (OR: 1.09; 95% CI: 1.01–1.17; *p* < 0.05), whereas replacement by MVPA was associated with a significantly lower likelihood of having high SO risk (OR: 0.47; 95% CI: 0.35–0.64; *p* < 0.05). We further determined whether the superior effect of MVPA compared to LPA on SO risk was reflected by differences in PA volume rather than intensity per se, by reallocating a PA volume block (20 000 daily accelerometer counts) derived from MVPA, with an equal block derived from LPA, while holding total PA volume constant. The analysis revealed a 15% higher SO risk (95% CI: 1.05–1.26; *p* < 0.05) when substituting equal PA volumes derived from MVPA with LPA.

### 3.3 SO Risk Across Levels of MVPA

**FIGURE 1:**
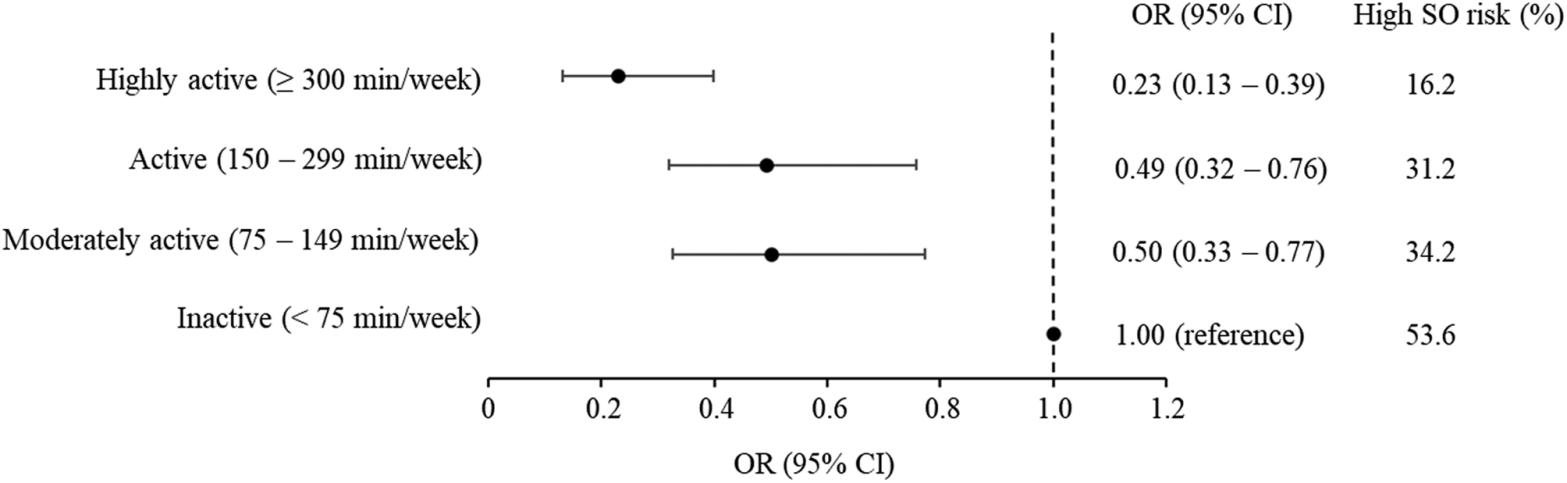
Odds ratios (OR) with 95% confidence intervals (CI) of high sarcopenic obesity (SO) risk across levels of time spent in moderate‐to‐vigorous physical activity (MVPA). Frequency (%) of participants with high SO risk is reported across levels of MVPA. Analyses are adjusted for age, sex, study centre, marital status, education years, smoking habits, general health status, number of disease/conditions related to musculoskeletal health, high‐sensitivity C‐reactive protein level, engagement in muscle‐strengthening activities, adherence to recommended protein intake, adherence to low‐risk consumption of alcohol, sedentary time and accelerometer wear time.

The likelihood of having a high SO risk across the four levels of time spent in MVPA is shown in Figure 1. Compared to the inactive group, a significantly (*p* < 0.05) lower likelihood of having a high SO risk corresponding to ≈50% to 80% was observed across all higher MVPA levels, including the moderately active group, with the largest risk reduction observed among the highly active group (Figure 1). Further, participants in the highly active group (≥ 300 min/week) had a significantly lower likelihood of high SO risk (OR: 0.50; 95% CI: 0.33–0.77; *p* < 0.05) compared to those in the active group (150–299 min/week). The particular benefit of belonging to the highly active group was also indicated by the substantially lower proportion of high SO risk participants shown in Figure 1. In contrast, no difference was evident when comparing the odds of having high SO risk between participants classified as moderately active and those in the active group (OR: 0.97; 95% CI: 0.65–1.45; *p* < 0.05).

### 3.4 SO Risk Across Levels of LPA

**FIGURE 2:**
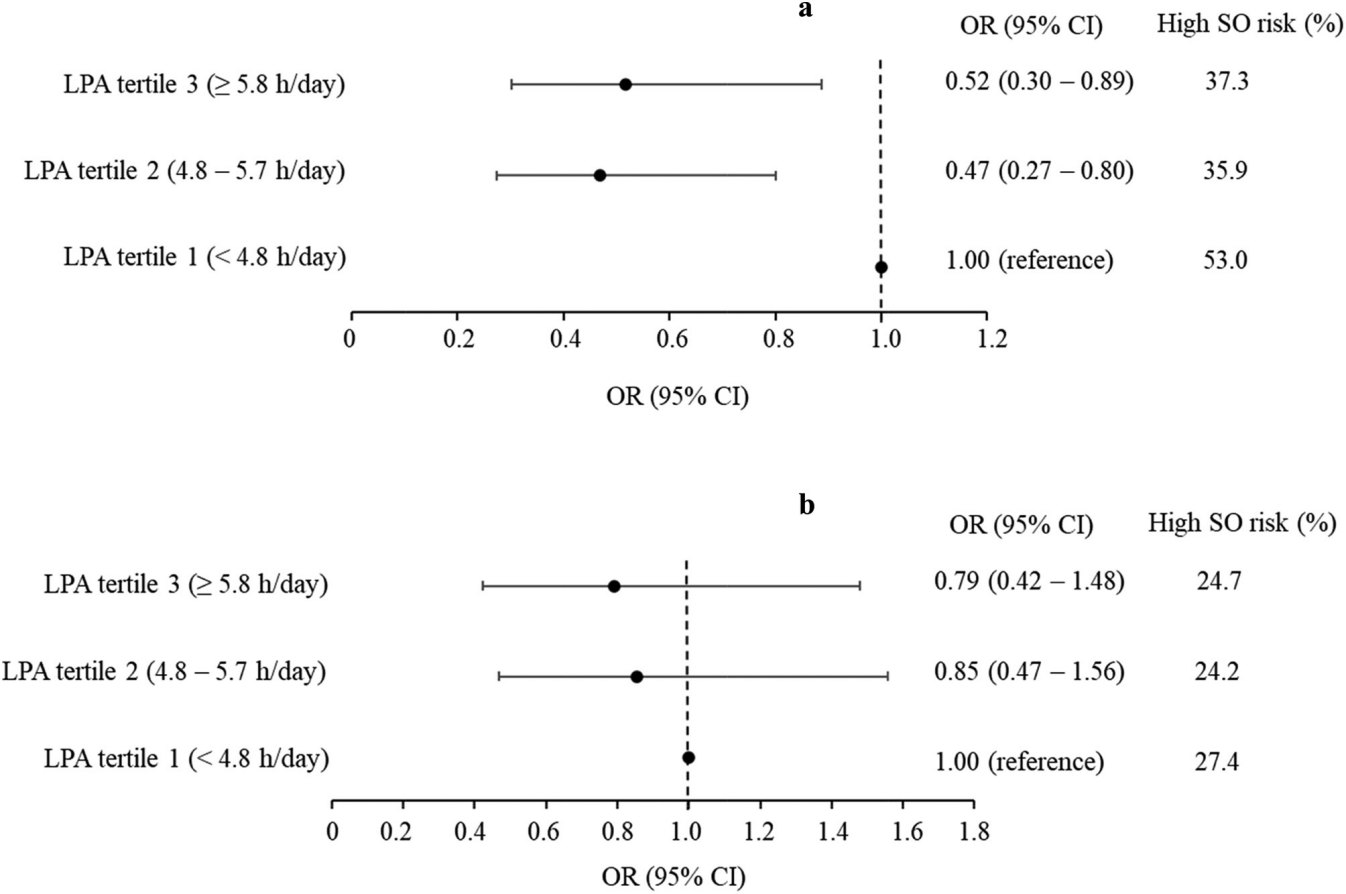
Odds ratios (OR) with 95% confidence intervals (CI) of high sarcopenic obesity (SO) risk across tertiles of daily time spent in light physical activity (LPA) in older adults below (a) and above (b) the recommended minimum amount of moderate‐to‐vigorous physical activity (MVPA). Frequency (%) of participants with high SO risk is reported across LPA tertiles. Analyses are adjusted for age, sex, study centre, marital status, education years, smoking habits, general health status, number of disease/conditions related to musculoskeletal health, high‐sensitivity C‐reactive protein level, engagement in muscle‐strengthening activities, adherence to recommended protein intake, adherence to low‐risk consumption of alcohol, time in MVPA and accelerometer wear time.

We further sought to determine whether LPA influenced SO risk independently of adherence to the MVPA guideline (≥ 150 min/week). The results are shown in Figure 2a,b. In participants not adhering to the MVPA guideline, belonging to either the 2nd or 3rd LPA tertiles was associated with a significantly lower likelihood (≈50%, *p* < 0.05) of having high SO risk compared to those in the 1st LPA tertile (Figure 2a). Notably, no significant difference in the likelihood of having high SO risk was evident between the 2nd and 3rd LPA tertiles (OR: 0.94; 95% CI: 0.54–1.65; *p* = 0.83). Finally, no impact on SO risk was observed across LPA tertiles among participants adhering to the MVPA guideline (Figure 2b).

## 4 Discussion

The findings of the present study suggest that engagement in MVPA, even below the minimum recommended amount, is sufficient to reduce SO risk. Moreover, older adults who accumulate at least twice the minimum recommended amount of MVPA benefit from the greatest risk reduction. These findings were independent of adherence to guidelines for adequate protein intake and levels of systemic inflammation. Furthermore, time in LPA was inversely related to SO risk in older adults who did not meet the MVPA guideline, highlighting the importance of promoting increased PA levels, regardless of intensity, for preventing SO in sedentary older adults.

Several studies have previously shown that higher PA levels are related to a lower prevalence of SO [6, 7, 8], which aligns with the findings of the present study. Notably, based on self‐reported time in MVPA, previous studies have shown that belonging to the highest PA category seems necessary to benefit from lower SO risk with potential benefits of belonging to the moderate compared to the low PA category left inconclusive [6, 7, 8]. In contrast, our data shows that compared to the most inactive group (< 75 min/week of MVPA), participants with weekly amounts of MVPA between 75 and 149 min/week had a significantly lower likelihood of having a high SO risk, even though the amount of MVPA was insufficient in relation to the MVPA guideline of ≥ 150 min/week. The different PA thresholds related to a lower SO risk reported previously compared to the present study may partly be explained by the use of self‐reported estimations of weekly PA time in previous studies, which are likely to become overestimated compared to accelerometer‐determined MVPA time [30]. Interestingly, a previous study based on accelerometry‐determined PA data has shown that the risk of sarcopenia was substantially lower in older adults accumulating 15 to 20 daily minutes of MVPA per day compared to lesser amounts [31], which strengthens the findings of the present study. Although the exact shape of the dose–response relationship between MVPA time and SO risk is yet to be determined, our finding suggests that MVPA is beneficially associated with a lower SO risk with continuing benefits for highly active participants engaging in MVPA beyond the minimum recommended amount (Figure 1). Indeed, those belonging to the highly active group (≥ 300 min/week) had a lower likelihood of having a high SO risk compared to those engaging in MVPA within a range of 150–299 min/week as targeted by the MVPA guideline. This finding is also in line with previous research investigating effects beyond recommended amounts of MVPA on indicators of physical function, which comprise components closely related to SO risk, revealing a better functional capacity among highly active older adults (≥ 300 min/week) compared to those who are active (150–299 min/week) [32]. Regarding the role of MVPA on SO risk, the fact that a hypothetical replacement of a 30‐min time period of LPA with MVPA was related to a lower SO risk supports current PA guidelines endorsing daily time in MVPA for optimal health benefits. This was further evidenced by the finding that replacing a fixed PA volume derived from MVPA with an equal volume derived from LPA was associated with a lower SO risk.

Importantly, reallocating a given time period of SB with LPA was related to a lower SO risk, suggesting a beneficial impact of lower intensity PA on SO risk. This finding supports previous research that has shown links between LPA and metabolic syndrome risk [14] and sarcopenia risk [33] in older adults. Interestingly, the beneficial impact of more daily time in LPA on the likelihood of having high SO risk was observed in older adults who do not adhere to the MVPA guideline (< 150 min/week). Therefore, targeting increased daily time in PA, regardless of intensity may be a feasible strategy to increase total PA volume and reduce SO risk in sedentary older adults. In contrast, no corresponding impact of LPA on SO risk was observed among older adults adhering to the MVPA guideline. The two studies that previously analysed the association between objectively measured time spent in LPA and SO risk reported the association to be either non‐existent [9] or weak [10], where adjustment by time in MVPA largely removed the association in the latter study. Although these studies did not specifically consider the moderating effects by adherence to the MVPA guideline in their analyses, their results are in line with our finding suggesting that certain amounts of MVPA will infer impacts on SO risk that are superior to any effects inferred by LPA. The fact that the frequency of participants with high SO risk was substantially lower across tertiles of LPA among those adhering to the MVPA guideline compared to those who did not (Figure 2a,b), further supports this notion.

The fact that levels of systemic inflammation were considered in our analyses enables more insight from our study findings, suggesting that a physically active lifestyle can influence SO risk in older adults at different levels of systemic inflammation. Although the interplay between systemic inflammation and the development of SO is not fully clarified, several pro‐ and anti‐inflammatory circulating markers have previously been associated with DXA‐derived body composition markers [34], and C‐reactive protein levels have been positively related to fat mass and inversely associated with ALM [35]. A potential mechanism underlying the favourable effect of MVPA on SO risk has been indicated in a previous study showing an inverse relationship between time in MVPA and CRP level in older women [36], suggesting the potential of PA to act on important factors related to SO development. Also, increased PA level may reduce SO risk by promoting a healthy energy balance counteracting accumulation of excess body fat, and mitigating decline in dimensions of physical function (e.g., strength, endurance and balance) during ageing.

### 4.1 Strengths and Limitations

This is the first study that investigates associations between objectively assessed time in PA and SO risk in older men and women, while accounting for several important biological, clinical and behavioural factors. For example, analyses were adjusted by adherence to guidelines for adequate intakes of dietary proteins for older adults, which otherwise may confound associations between PA and SO risk. Indeed, previous research has shown that intakes of dietary proteins are positively associated with lean mass [37, 38] and sarcopenia risk [16], highlighting the importance of dietary proteins to support healthy muscle ageing [29].

Another strength of the current study is the use of accelerometers, which is an extensively validated tool for assessing time in PA of different intensities in free‐living settings [12]. However, a limitation related to accelerometry is that the monitor cannot correctly capture all types of activities, as the ability to determine changes in energy cost of activities relies on proportional changes in bodily accelerations. For example, walking a given distance with or without a heavy backpack will yield different energy costs without being reflected by proportional changes in bodily accelerations. For this reason, we additionally collected self‐reported information about engagement in MSA, which includes resistance‐type exercises that may be less well captured by the accelerometer. Furthermore, given that resistance‐type exercises have well‐established effects on muscular hypertrophy [39], being able to differentiate between whether time spent in MVPA is mainly based on aerobic or resistance‐type exercises allows for deeper insight into the role of PA behaviours on SO risk. For example, the fact that observed effects on SO risk across levels of adherence to the MVPA guideline remain after adjustment for weekly engagement in MSA, suggests that beneficial influences on SO risk are not limited to resistance‐type activities, thus supporting the MVPA guideline endorsing accumulation of aerobic‐type MVPA. However, further research is warranted to determine how different types of PA impact SO risk.

The study sample of older adults is limited to individuals who were non‐frail and did not fulfil diagnostic criteria for SO as stated by ESPEN and EASO [1]. Therefore, our sample is unlikely to be representative of wider populations of older adults with varying health status, including the presence of SO and frailty. Notably, older adults who fulfil diagnostic criteria for SO, with accompanying functional impairments, may accumulate less time in MVPA simply as a result of SO rather than being a factor for its development. Consequently, investigating associations between PA and SO risk in participants free of SO may reduce the risk of reverse causation. However, the cross‐sectional analysis of the current study hampers conclusions about the direction of observed associations. Therefore, future investigations based on longitudinal study designs are warranted to confirm the importance of PA behaviours in the prevention of SO in ageing populations. This would inform the design and implementation of successful PA interventions for the prevention and management of SO in clinical and public health settings.

## 5 Conclusion

PA is strongly associated with a lower likelihood of having a high SO risk in older adults, independently of the level of systemic inflammation and intakes of dietary proteins. Engagement in MVPA, even below the minimum recommended amount of 150 weekly minutes, is likely to reduce SO risk, while the greatest risk reduction requires at least twice the recommended amount. Light PA is related to SO risk in sedentary older adults, which supports the promotion of PA regardless of intensity for mitigating SO.

## Ethics Statement

Participants received oral and written information about the study and signed a written informed consent. All study procedures were conducted in accordance with the Declaration of Helsinki. The study was approved by local ethical review boards: Independent Ethics Committee of the Sant'Orsola ‐ Malpighi Hospital Bologna (Italy‐03/2011/U/Sper), Bioethics Committee of the Polish National Food and Nutrition Institute (decision of 03.04.2012), the Wageningen University Medical Ethics Committee (Netherlands‐11/41) and the National Research Ethics Committee East of England (UK‐12/EE/0109). All authors certify that they comply with the ethical guidelines for authorship and publishing in the *Journal of Cachexia, Sarcopenia and Muscle*.

## Conflicts of Interest

The authors declare no conflicts of interest.

## Supporting information


**Table S1:** Cutpoints used for classification of high sarcopenic obesity risk in the study sample.
