# Supplementary material for: Accelerometer‐Determined Physical Activity and Sarcopenic Obesity Risk in Older European Men and Women
Source: J Cachexia Sarcopenia Muscle. 2025 Dec 4;16(6):e70149. doi: 10.1002/jcsm.70149 (PMC12678005; doi:10.1002/jcsm.70149)
Supplement: Supplementary file 1 — Table S1: Cutpoints used for classification of high sarcopenic obesity risk in the study sample. [file JCSM-16-e70149-s001.docx]

Supplementary table 1. Cutpoints used for classification of high sarcopenic obesity risk in the study sample.

|  | Men (n=361) | | Women (n=501) |
| --- | --- | --- | --- |
|  |  | |  |
| Waist circumference (cm) | ≥94 | | ≥80 |
| ALM (%BW) | <30.5 | | <24.4 |
| Handgrip strength (kg) | <40.3 | | <25.2 |
| 5-STS (s) | >9.2 | | >10.2 |
|  |  |  | |

^Abbreviations: ALM, Appendicular lean mass; BW, body weight, 5-STS, 5-times sit-to-stand test.^
